# Resorbable GBR Scaffolds in Oral and Maxillofacial Tissue Engineering: Design, Fabrication, and Applications

**DOI:** 10.3390/jcm12226962

**Published:** 2023-11-07

**Authors:** Seyed Ebrahim Alavi, Max Gholami, Hasan Ebrahimi Shahmabadi, Peter Reher

**Affiliations:** 1 School of Medicine and Dentistry, Griffith University, Gold Coast, QLD 4215, Australia; ebrahim.alavi@griffithuni.edu.au (S.E.A.); max.gholami@griffithuni.edu.au (M.G.); 2 Immunology of Infectious Diseases Research Center, Research Institute of Basic Medical Sciences, Rafsanjan University of Medical Sciences, Rafsanjan 7717933777, Iran; ebrahimi@rums.ac.ir

**Keywords:** 3D printing, artificial intelligence, bone regeneration, polymer, resorbable membrane, tissue engineering

## Abstract

Guided bone regeneration (GBR) is a promising technique in bone tissue engineering that aims to replace lost or injured bone using resorbable scaffolds. The promotion of osteoblast adhesion, migration, and proliferation is greatly aided by GBR materials, and surface changes are critical in imitating the natural bone structure to improve cellular responses. Moreover, the interactions between bioresponsive scaffolds, growth factors (GFs), immune cells, and stromal progenitor cells are essential in promoting bone regeneration. This literature review comprehensively discusses various aspects of resorbable scaffolds in bone tissue engineering, encompassing scaffold design, materials, fabrication techniques, and advanced manufacturing methods, including three-dimensional printing. In addition, this review explores surface modifications to replicate native bone structures and their impact on cellular responses. Moreover, the mechanisms of bone regeneration are described, providing information on how immune cells, GFs, and bioresponsive scaffolds orchestrate tissue healing. Practical applications in clinical settings are presented to underscore the importance of these principles in promoting tissue integration, healing, and regeneration. Furthermore, this literature review delves into emerging areas of metamaterials and artificial intelligence applications in tissue engineering and regenerative medicine. These interdisciplinary approaches hold immense promise for furthering bone tissue engineering and improving therapeutic outcomes, leading to enhanced patient well-being. The potential of combining material science, advanced manufacturing, and cellular biology is showcased as a pathway to advance bone tissue engineering, addressing a variety of clinical needs and challenges. By providing this comprehensive narrative, a detailed, up-to-date account of resorbable scaffolds’ role in bone tissue engineering and their transformative potential is offered.

## 1. Introduction

The oral and maxillofacial area holds significance due to its multifaceted functions and aesthetic importance within the human body. This region possesses intricate anatomy and various tissue types [1]. Repairing bone defects resulting from trauma, tumours, or inflammation in these regions presents a significant challenge [2]. One widely used method for promoting bone formation is guided bone regeneration (GBR), which is extensively employed to treat mouth and facial area defects, particularly in dental implantology [2]. GBR is utilized as a regenerative technique to enhance ridge volume [3]. Its objective is to selectively attract osteogenic cells to the site of bone defects while excluding cells that might hinder osteogenesis [3]. Therefore, the primary objective of GBR is to establish a specific path for osteoblasts to access the osteogenic region using a barrier membrane. This allows the creation of fresh bone within this space, facilitated by the use of bone replacement materials as a framework. This process ultimately establishes a robust osteogenic environment [4]. If the stability of the osteogenic area is insufficient, external tissue pressure can lead to its collapse, causing the displacement of bone grafts and failure to achieve the desired clinical result [5,6]. Hence, an ideal barrier membrane material for GBR should find a balance between strong biocompatibility and excellent support [7].

GBR membranes are categorized into two main groups according to their degradation behaviour: non-resorbable (N-RES) and resorbable (RES) membranes. N-RES membranes need a subsequent surgical procedure to be taken out, posing an increased risk of problems like membrane exposure [8]. Conversely, RES membranes are metabolized by the body, lessening patient discomfort by removing the necessity for another surgical procedure [3].

Meanwhile, the scaffold material assumes a pivotal role in tissue regeneration, providing an environment conducive to cell adhesion, proliferation, and differentiation [9,10]. Furthermore, functional scaffolds can serve as carriers for local drug delivery, holding therapeutic agents to facilitate tissue healing [11]. Delivery systems are able to enhance the therapeutic effects of compounds [12,13,14,15,16,17,18,19,20,21]. Scaffolds featuring interconnected pores tend to foster greater bone growth compared to those with closed or absent pores. This is attributed to the improved delivery of osteoprogenitors when vascular ingrowth is facilitated [22]. Given the complex anatomical and microbiological context, the prevention of postoperative infections is a crucial imperative in the field of oral and maxillofacial surgery. Therefore, in order to ensure successful surgical outcomes, the advancement of resorbable GBR scaffolds depends on enhancing both their mechanical and antibacterial properties [23].

This paper aims to present a comprehensive review of oral tissue regeneration and GBR, emphasising the significance of resorbable scaffolds in GBR. Additionally, the review explores the application of three-dimensional (3D) printed scaffolds that can support graft materials and sustain bone regeneration in the context of implantology and explicitly [24,25].

## 2. Principles of Guided Bone Regeneration and Scaffold Requirements

GBR is a surgical method whereby bone grafts and barrier membranes are used together to repair minor imperfections near dental implants. Generally, this technique is utilized to treat dehiscence or fenestration defects that are ≥2 mm in dimension, with larger defects potentially requiring a combination with autogenous bone [26,27]. The underlying principle of GBR is to utilize the barrier membrane as a physical barrier to prevent the intrusion of rapidly growing epithelial cells and fibroblasts from the surrounding tissues into the bone defects. Instead, it allows only osteogenic cells from the adjacent bony walls to populate and regenerate the defect [28]. Advancements in medical and material science, particularly the incorporation of tissue engineering techniques in the early 1990s, have led to significant enhancements in GBR methodologies [2]. Over time, GBR approaches have evolved to include various elements like scaffolds, barrier materials, regenerative cells or stem cells, and cytokines or GFs [2]. Among these elements, the core components of GBR involve the utilization of GBR materials or combinations involving materials and cells [29]. These materials play a crucial role in GBR by serving as a barrier, preventing the infiltration of connective tissue, and creating a conducive environment for bone regeneration [2,30]. Moreover, the ideal biomaterials used in bone regeneration should possess a range of biological functions that enhance the inherent self-healing capabilities of bone tissue [2,31,32]. These functions encompass: (a) Supplying essential structural, compositional, and biochemical cues necessary for the creation of the new tissue, (b) Facilitating the recruitment, growth, and differentiation of progenitor cells, (c) Involving the host’s native immune cells to actively participate in the regenerative process, (d) Restoring a sufficient local blood supply to support bone healing and remodelling, and (e) Exhibiting anti-infective properties, especially in non-sterile contexts such as bone resorption resulting from conditions like periodontitis [2,27,31,32]. Even with the rise of scaffold-free tissue engineering, which utilises cell sheets, spheroids, and tissue strands as the foundation, traditional biomaterial scaffolds remain the preferred method for bone regeneration. This is because of their favourable degradation characteristics, beneficial mechanical properties, and their ability to carry and release vital biomolecules, such as growth and differentiation factors, with controlled precision [33].

Scaffolds are specialised 3D biomaterials designed with porosity, fibrous structures, or permeability. They facilitate cell interactions, support cell viability, and promote the deposition of the extracellular matrix (ECM) during tissue regeneration. Importantly, these scaffolds are engineered to minimise inflammation and toxicity while also undergoing controlled biodegradation [34]. Generally, an optimal scaffold material needs to possess several key characteristics. Firstly, it should be biocompatible. Secondly, it should have controllable degradation. Thirdly, the scaffold should possess appropriate physico-chemical features to closely mimic the structure of the original tissues’ ECM [9]. Moreover, the ideal scaffold should have the capacity to blend different materials with specific functions, accomplished through engineered surface modifications, cell encapsulation techniques, and controlled release of chemicals [9,35].

Furthermore, the scaffold should actively promote and regulate specific events occurring at both the cellular and tissue levels, ensuring proper tissue development and regeneration [36]. In scaffold-based tissue engineering, the scaffold plays multiple crucial roles. Firstly, it must offer sufficient mechanical strength and stiffness to mimic the mechanical properties of the damaged or absent tissue it is replacing [9]. Additionally, a successful scaffold should not only facilitate the initial growth of new tissue but also support the ongoing maturation and remodelling processes. It should provide the necessary support and appropriate morphology to guide the developing tissue as it matures [37]. Furthermore, the design of the scaffold should take into consideration its degradation kinetics, meaning how it breaks down over time, and its physico-chemical features [9].

In summary, the scaffold’s functions encompass providing mechanical strength, promoting tissue growth and maturation, and accounting for degradation kinetics and physico-chemical characteristics, all of which are vital for the success of tissue engineering endeavours.

### 2.1. Guided Bone Regeneration Technique and Its Role in Implantology

Dental implantology relies on implant prosthesis as a crucial method to address dentition defects. However, in clinical surgeries, a common challenge is encountering insufficient alveolar bone volume, caused by factors like periodontitis and local alveolar process absorption [38]. To tackle this issue, various approaches such as distraction osteogenesis, autografts, and GBR have been employed [39]. Despite its potential, distraction osteogenesis may lead to unwanted tissue scars due to its immature development [38]. While autogenous bone grafting has a well-established clinical history and evident alveolar augmentation benefits, addressing the issues of graft damage and repair is of the utmost importance [38]. In contrast, the GBR technique utilizes a barrier membrane to obstruct the infiltration of fibrous tissue and create a favourable local environment for bone regeneration, solidifying its position as one of the most efficacious strategies for enhancing alveolar bone volume in the field of dental implantology [40]. This membrane can also function as a carrier for GFs like bone morphogenetic protein-2 (BMP-2), insulin-like growth factor (IGF), and other factors associated with bone development [41,42]. Apart from its barrier function, the membrane plays several essential roles in the GBR process. It safeguards the integrity of the blood clot in the treated area, aids in the transportation of oxygen and nutrients, and facilitates the establishment of microcirculation [38]. Additionally, Omar et al. [42] suggested that the membrane actively hosts and modulates the molecular activities of membrane-associated cells during GBR, further enhancing its significance [42]. Hence, it is evident that the choice of GBR membrane significantly influences the therapeutic outcome of GBR surgery [38].

### 2.2. Key Considerations for an Ideal Resorbable Scaffold

Scaffold-based biomimetic bone replacements aim to replicate bone’s structural, mechanical, and biological attributes to replace missing tissue. For bone substitutes employed to treat significant segmental defects, they need to encourage osteoinduction, osteoconduction, and osseointegration [43]. Osteoinduction involves prompting pluripotent precursor cells to differentiate into bone-forming osteoblasts [43,44]. Osteoconduction encompasses aiding growth on the scaffold’s surface and within its pores or channels through processes like cell adherence, proliferation, and the creation of a new ECM [45]. Osseointegration, defined as the proper establishment of a mechanically stable direct connection between bone tissue and the implanted material, should transpire without the growth of fibrous tissue [46].

Several scaffold properties, including material composition and spatial organisation, must be thoughtfully balanced and considered to achieve this. When used in regenerative techniques, scaffold materials should demonstrate adequate biocompatibility [43]. In contrast to procedures like joint replacement, regenerative strategies require scaffold biodegradability, as the central goal is to encourage and facilitate the inherent healing of tissue [47]. Consequently, an optimal scaffold material for tissue regeneration needs to be completely degradable over time and progressively substituted by a naturally formed bone matrix within the body. The rate of GBR scaffold resorption is influenced by a number of complicated interactions, including the scaffold’s composition, the rate of disintegration, and the tissue environment. Enzymatic cleavage and passive hydrolysis are the two main processes that drive this process. When collagen or other naturally occurring polymer-based scaffolds are employed, degradation predominantly takes place through highly specialized enzyme cleavage. Due to the susceptibility of these polymers to enzyme action within the body, precise breakdown in conjunction with natural tissue regeneration is made possible. Passive hydrolysis is crucial in the case of scaffolds made of synthetic polymers. Under physiological settings, these polymers eventually degrade, influenced by factors like molecular weight, chain structure, comonomer ratio, residual monomer content, crystallinity, annealing, and sterilization techniques. In order to ensure their absorption without unfavourable biological effects and to support successful tissue regeneration, scaffold design must take into account the complex interplay of these mechanisms, which has a profound impact on the resorption rate [48,49]. A fundamental difficulty in GBR for oral and maxillofacial abnormalities is to achieve a precise balance between the rate of scaffold degradation and the rate of tissue regeneration. Regenerated tissue can take its place after resorbable scaffolds gradually disappear. But coordinating the precise synchronization of these activities is a complex endeavour dependent on a number of variables. The intrinsic variety of patient responses presents a significant difficulty because each person may exhibit unique differences in their capacity for tissue regeneration, making it difficult to estimate the precise rate of tissue regrowth. In this delicate balance, the scaffold’s material characteristics play a crucial role. The best material must be chosen for the particular clinical situation, since various materials, such as synthetic polymers, natural polymers, and ceramics, resorb at different rates [50,51]. Furthermore, the scaffold should provide adequate mechanical reinforcement. To achieve this, material characteristics such as compressive strength, stiffness, and elasticity should align with those characteristics of bone during the specific stage of regeneration [43,52,53]. For instance, during endochondral ossification, the biomechanical context is marked by a relatively low Young’s modulus of around 8 kilopascal (kPa), whereas fully developed healthy bone tissue exhibits values in the gigapascal (GPa) range (0.1 to 2 GPa for trabecular bone and between 15 and 20 GPa for cortical bone) [54,55].

The scaffold material being porous is critical in bone regeneration, as it should allow for cell reorganization and vascularization. Consequently, optimizing factors such as porosity, pore size, and interconnectivity become vital while still meeting the mechanical prerequisites. A pore size of approximately 100 µm has been identified as beneficial for cell migration, encouraging the initial phases of bone formation involving cell recruitment, proliferation, differentiation, and the formation of the ECM [43]. However, larger pore sizes are necessary for effective bone tissue development to facilitate vascularisation. Hence, optimal outcomes in tissue regeneration could be achieved by utilising multiscale porous scaffolds incorporating both small and large pores [43,56]. Woodard et al. [57] conducted an assessment of the osteoconductivity of hydroxyapatite scaffolds with multi-scale porosity, comparing them to scaffolds featuring a single pore size. The non-microporous (NMP) scaffolds exclusively possessed macroporosity ranging from 250 to 350 μm, while the microporous (MP) scaffolds featured both macroporosity and microporosity ranging from 2 to 8 μm. The results demonstrated that, after 8 weeks, only the MP scaffolds contained bone tissue. Furthermore, the results underscored the effectiveness of MP scaffolds as carriers for drug delivery [57]. Moreover, a substantial level of interconnectivity among the pores is crucial to ensure proper cell dispersion, attachment, and the ingrowth of host blood vessels. Furthermore, cell attachment and subsequent intrinsic scaffold growth is dependent on its surface and internal configurations [58].

#### 2.2.1. Biocompatibility and Cell Interaction

Biocompatibility refers to a biomaterial’s ability to serve its intended role in medical treatment without inducing undesirable local or systemic reactions in the recipient. This encompasses achieving the most suitable clinically significant performance [43] and applies to the material’s overall structure and potential degradation products. Specifically, regarding scaffold materials, biocompatibility entails supporting cell survival and maintaining specific cellular functions relevant to the targeted tissue type while preventing cell apoptosis or triggering immune responses [43,59].

Tissue engineering using GBR can leverage a promising combination of osteoconductive scaffolds, stem cells, and GFs for bone regeneration [60]. Scaffolds allow the transportation of nutrients, waste materials, and regulatory signals necessary for cell proliferation and differentiation [61]. Additionally, under specific culture conditions, scaffolds can be controlled in their degradation rate, and studying the interactions of degraded molecules or ions with cells holds significant value [61].

#### 2.2.2. Mechanical Strength and Stability

The mechanical characteristics, including tensile strength, elastic modulus, and stiffness are critical requirements for scaffolds, as they determine the scaffold’s structural roles and long-lasting nature. In an ideal scenario, the scaffold should be tailored to correspond to the anatomical location and imitate the inherent composition of cancellous bones or tissues [62]. Evaluation methods, such as compressive and tensile tests, are essential for assessing the scaffold’s mechanical strength. For bone implants, possessing a favourable set of mechanical characteristics is essential for promoting tissue remodelling and avoiding the adverse effects of stress shielding. Dissimilar properties between the bone and the implant material can result in stress-shielding, causing adaptive alterations in bone strength and stiffness [63].

The healing rate of bone tissue varies with age, and this is an important consideration when designing scaffolds for orthopaedic applications. In younger individuals, bone fractures usually require up to six weeks before weight-bearing is possible, and about a year is needed for complete fracture integration. Nonetheless, the healing process can be notably lengthier for older individuals [64]. Striking the right equilibrium between optimal mechanical attributes and structural functionality in scaffolds presents a substantial challenge for researchers. Although many scaffolds have exhibited promising mechanical traits in simulated laboratory settings, they often encounter failures during real-world integration due to inadequate vascularization capacity. As a result, attaining effective vascularization and cell infiltration necessitates maintaining a delicate equilibrium between the mechanical properties and the porous architecture during the development of scaffolds [65].

In tissue engineering, scaffolds have traditionally been developed using both natural and synthetic materials. Each type of material has its own limitations. To overcome these challenges, scientists have investigated the concept of blending two or more materials to leverage their individual advantageous traits [63,66]. For example, Chong et al. [66] utilized the electrospinning technique to directly deposit polycaprolactone (PCL)/gelatine nanofibers onto polyurethane wound dressings. The effectiveness of the resulting composite, referred to as TG-NF, in comparison to PCL/gelatine nanofibers, regarding the proliferation of human dermal fibroblast (HDF) cells, was assessed. The findings revealed that TG-NF led to a 4.8% increase in HDF cell proliferation when compared to PCL/gelatine nanofibers. Natural materials are known for their excellent biological activity, strong biocompatibility, and hydrophilic nature. However, they often suffer from insufficient mechanical characteristics and unpredictable degradation rates [63,67]. Conversely, synthetic polymers offer significant mechanical strength and ease of processing, but they lack surface features that promote cell identification, resulting in reduced cell adhesion ability and hydrophobic behaviour [63,68].

#### 2.2.3. Porosity and Permeability

Enhanced permeability of scaffolds has a beneficial effect on bone ingrowth, while concurrently deterring the development of cartilaginous tissue at the regenerated site [69]. Various factors, including porosity, orientation, size, distribution, and interconnectivity of pores, impact permeability. Larger pore sizes are advantageous for cell growth and proliferation, as they remain open for an extended duration during ongoing growth. This aids in enhanced nutrient and oxygen supply, thus promoting vascularization in newly formed bone tissues [70]. Notably, O’Brien and colleagues carried out an in vitro study in which they decreased the pore size of collagen–glycosaminoglycan scaffolds, leading to a reduction in permeability [69]. Surprisingly, they found that the smallest pore size resulted in the highest seeding efficiency [69]. The interconnectivity of pores is also a vital factor in ensuring proper permeability and preventing premature pore occlusion [71]. Adequate interconnectivity within porous scaffolds is essential for facilitating effective cell infiltration. As an example, a coralline scaffold based on ceramic with a pore size of 500 µm displayed favorable cell infiltration [69]. The scaffold’s porosity directly affects cell attachment, degradation rate, and the release of carriers by influencing the surface area of interactions between cells and the scaffold [58]. The high porosity of the structure enables cells to move throughout the scaffold’s entire length and settle at its base without adhering to surface proteins [72]. Conversely, when the pore size is restricted and there is inadequate room for infiltration, cells are driven to differentiate rather than multiply [69]. As a result, smaller pore sizes may not promote bone formation effectively, as they could lead to a hypoxic environment and trigger chondrogenesis instead of osteogenesis [73].

## 3. Addressing Challenges in Guided Bone Regeneration with Resorbable Scaffolds

While GBR is typically dependable for bony defects, its effectiveness becomes restricted and uncertain when applied to vertical extra-cortical bone augmentation, resulting in a notable occurrence of complications and failures (exceeding 20%) [74]. These challenges can be linked to the utilization of barrier membranes, which are presently classified as N-RES and RES [75]. N-RES membranes such as polytetrafluoroethylene, expanded polytetrafluoroethylene membrane, and titanium mesh necessitate a secondary surgical intervention for removal and frequently contribute to soft tissue dehiscence. This often results in wound infections and protracted healing durations [74].

GBR without the use of a barrier membrane, which involves the synthetic scaffolds without a barrier membrane, is regarded as a favourable method for bone augmentation due to the significant issues linked to resorbable barrier membranes. RES membranes like polylactide, polyglycolide, PCL, and collagen exhibit diminished volume stability during bone healing due to faster resorption, ultimately leading to the premature decline of mechanical attributes. Additionally, the biodegradation of these membranes triggers an inflammatory response in the surrounding soft tissue [74,76].

Scaffolds such as osteoconductive bone substitutes, collagen, Poly (lactic-co-glycolic acid) (PLGA), and others, play an essential part in GBR and the healing of critical-size bone defects [2]. These scaffolds not only uphold the required space but also encourage bone development by facilitating the movement of osteoblasts and the mineralization of the newly created bone matrix, akin to bioceramics containing calcium phosphate (CaP) [77]. Moreover, the existence of a scaffold within a bone regeneration site, regardless of whether a barrier membrane is used or not, serves to inhibit the migration of fibrous soft tissue. This migration could potentially undermine the healing of the defect [77].

### 3.1. Controlling Resorption Rate

Polymeric materials derived from natural proteins or minerals are extensively investigated for their role in intraoral bone regeneration. These scaffolds imitate the structural framework of natural bone tissues and frequently replicate the makeup of natural bone, incorporating both organic and inorganic constituents [78]. Notably, collagen type I and alginate constitute fundamental organic polymeric structures within natural bone tissue, while hydroxyapatite and CaP minerals constitute the predominant naturally occurring inorganic elements in bone [79]. Indeed, organic biopolymers sourced from nature are extensively favoured as scaffold materials for bone regeneration due to their exceptional biocompatibility, biodegradability, and osteoconductivity. These materials readily interact with vital growth factors (GFs) like bone morphogenetic proteins (BMPs), amplifying their regenerative potential.

**Figure 1:**
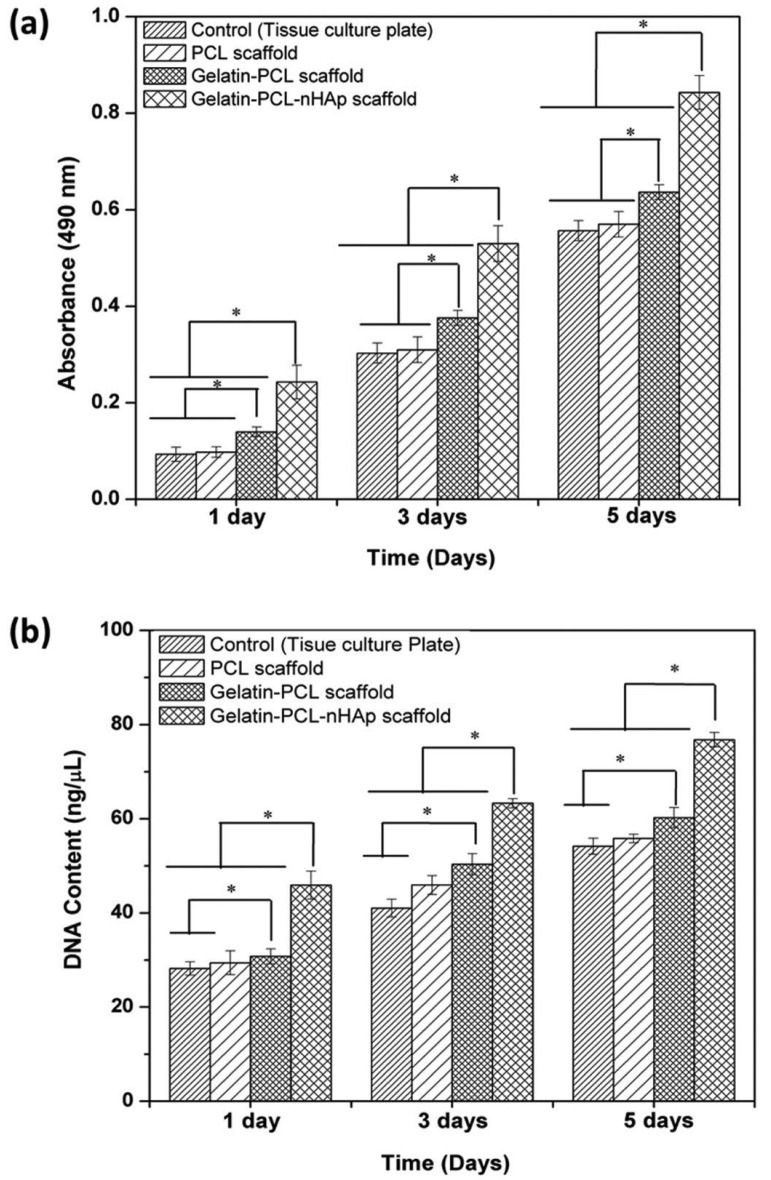
(**a**) The MTT assay and (**b**) DNA quantification were conducted on human osteoblasts grown on different substrates: the control group (tissue culture plate), PCL, gelatine-PCL nanofibrous scaffold, and gelatine-PCL-nHAp (nHA) nanocomposite scaffold-20 min. (*) indicates a significant difference between the scaffolds (*p* < 0.05). Reproduced with permission from Ref. [81]. Copyright 2021 Elsevier, Amsterdam, The Netherlands.

Some researchers [79,80] have explored the amalgamation of these natural materials with other bone substitute grafts like beta-tricalcium phosphate (β-TCP) and hyaluronic acid (HA) to further amplify their efficacy [80,81]. Cai et al. [80] conducted a study exploring the potential of 3D electrospun PLGA/PCL scaffolds for enhancing dental tissue regeneration. In this study, pig dental epithelial (pDE) cells and human tooth pulp-derived dental mesenchymal (hDM) cells were utilized. The study also examined whether the incorporation of nano-hydroxyapatite (nHA) could promote dental cell differentiation. The findings revealed that the electrospun scaffolds possessed adequate porosity, ranging from 93.972 ± 1.170 to 99.476 ± 0.151 (*v*/*v*)%, facilitating robust cell infiltration and growth. Additional ultrasonic treatment resulted in a less uniform scaffold porosity, leading to noticeable cell clustering and improved interactions between hDM and pDE cells. Furthermore, the incorporation of nHA was found to enhance dental cell differentiation, with an increase of approximately 41.4% and 76.5% on day 28 for hDM and hDM + pDE cells, respectively. However, it is important to note that this also led to a reduction in fibre diameter, with measurements of 2.198 μm ± 0.396 and 0.810 μm ± 0.435 for 3D electrospun scaffolds treated with ultrasonics (3Du) and 3Du containing nHA (3DHu), respectively. Additionally, the scaffold porosity decreased to 99.476 ± 0.151 (*v*/*v*)% and 99.000 ± 0.298 (*v*/*v*)% on day 28 for 3Du and 3DHu, respectively, which inhibited cell infiltration and proliferation by approximately 65.1% and 91.6% on day 28 for hDM and hDM + pDE cells, respectively [80]. In summary, ultrasonically treated wet-electrospun PLGA/PCL scaffolds prove to be a suitable material for dental tissue engineering, paving the way for potential in vivo evaluations of this model [80]. In another study by Gautam et al. [81], an electrospun nanocomposite scaffold was fabricated for bone tissue engineering, incorporating gelatine, PCL, and nHA. The results demonstrated that the gelatine-PCL-nHA nanocomposite scaffold-20 min exhibited an average fibre diameter of 615 ± 269 nm and an average pore size of 4.7 ± 1.04 μm. Additionally, the presence of nHA particles was observed on the surface of the gelatine-PCL scaffold. The results of MTT assays and DNA quantification demonstrated that the inclusion of nHA into the nanocomposite scaffold enhanced the viability and proliferation of human osteoblast cells by 24.4% and 21.9%, respectively (Figure 1). Furthermore, the cell-scaffold constructs displayed effective cellular attachment with well-spread cells, showcasing the distinctive polygonal morphology typically seen in osteoblasts on the gelatine-PCL-nHA nanocomposite scaffold. Consequently, the in vitro analysis of the electrospun nanocomposite scaffold strongly indicates that the gelatine-PCL-nHA scaffold holds promise as a prospective candidate for applications in bone tissue engineering [81].

### 3.2. Graft Retention and Stability

Hydrogel scaffolds, defined by their elevated water content and hydrophilic polymer chains, possess distinct characteristics such as biocompatibility, elasticity, and the ability for chemical modification. They can imitate the ECM and serve as a growth medium for cells and tissues [82,83,84]. Due to these properties, hydrogels find widespread applications in biomedical research, including drug delivery and various regenerative medicine approaches, such as bone tissue regeneration [82,85]. To augment cell adhesion, specific peptide sequences like arginine-glycine-aspartic acid are incorporated into alginate hydrogels [86].

## 4. Materials and Fabrication Techniques for Resorbable Guided Bone Regeneration Scaffolds

**Figure 2:**
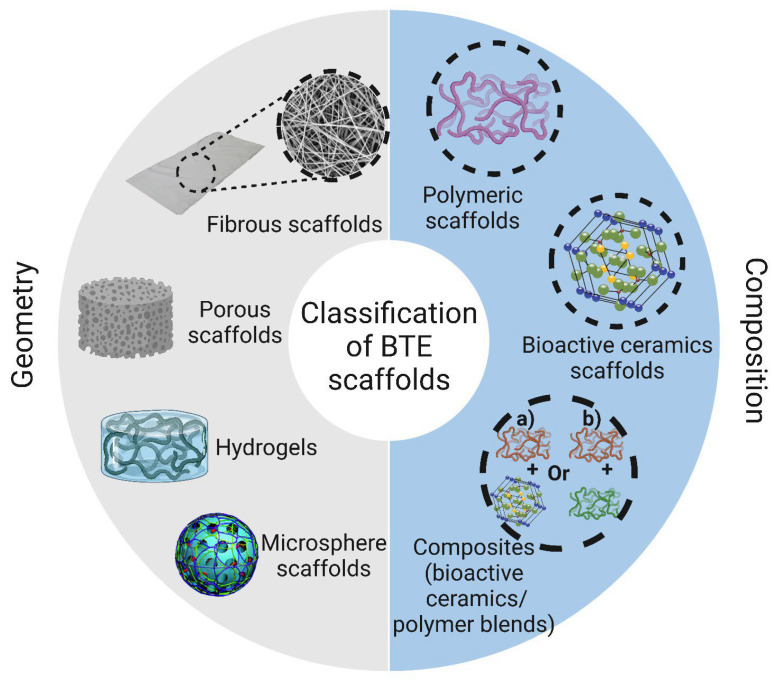
Classification of polymeric scaffolds employed in bone tissue engineering based on their geometry and makeup. Reproduced with permission from Ref. [87]. Copyright 2023 MDPI, Basel, Switzerland.

Bioresorbable scaffolds refer to materials that can be broken down into smaller components in the body, subsequently undergoing elimination through natural processes. This results in the complete elimination of the original initial material without causing any detrimental biological impacts [48]. The degradation process can occur through highly specific enzymatic cleavage, observed in natural polymers like collagen, or through passive hydrolysis, involving the breakdown of synthetic polymers under physiological conditions [48]. Multiple factors, such as molecular weight, chain configuration, comonomer ratio, residual monomer content, crystallinity, annealing and sterilization methods, as well as the inclusion of drugs or other additives, influence the degradation rate [49]. A significant challenge in developing bioresorbable materials is ensuring that the degradation rate aligns with the natural pace of tissue remodelling while also maintaining sufficient mechanical properties of the scaffold. Striking this balance is essential to avoid the possibility of scaffold visibility in periodontal surgery, primarily due to potential inflammation-related issues in the sensitive gingival tissues that cover the alveolar bone. It is worth noting that this risk is also applicable when utilising non-resorbable metallic scaffolds. To mitigate this, precise flap design and suturing techniques are crucial to achieve primary closure and promote regular wound healing [48]. In order to categorize the scaffolds used in bone tissue engineering, their geometry is typically taken into consideration. These options include fibrous scaffolds, porous scaffolds, hydrogels, and microsphere scaffolds. They can also be divided into groups according to their composition, which include composite materials made of bioactive ceramics and polymers, polymeric scaffolds, and scaffolds made of bioactive ceramics (Figure 2) [87].

### 4.1. Biodegradable Polymers for Scaffold Fabrication

**Figure 3:**
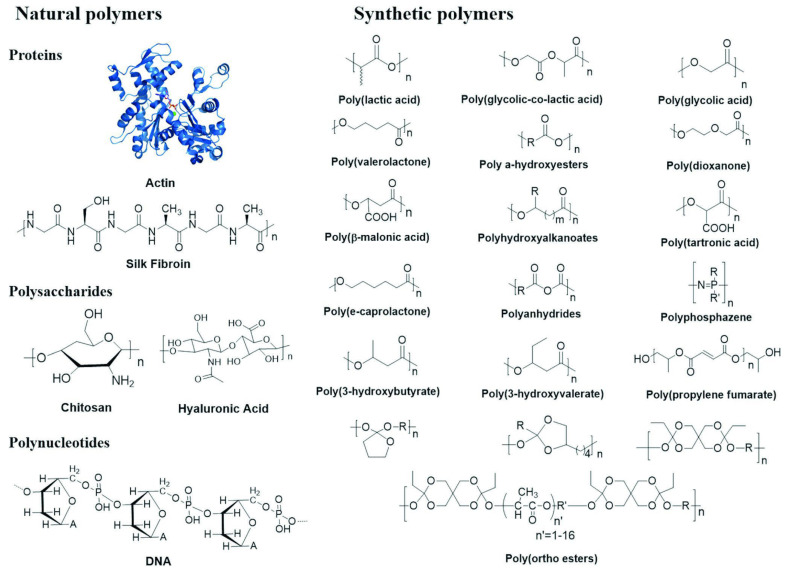
Various polymers for use as scaffold materials for bone tissue engineering. Reproduced with permission from Ref. [88]. Copyright 2020 Wiley-VCH GmbH, Weinheim, Germany.

Polymers play a significant role as scaffold materials in bone tissue engineering due to their biodegradability, which supports bone tissue regeneration and eventual absorption by the body (Figure 3) [88,89]. Due to their likeness to ECM constituents, natural polymers (e.g., collagen, chitosan, gelatine, silk fibroin, alginate, cellulose, and starch) provide exceptional biocompatibility, strong cell adhesion, and favourable conditions for cell growth [51,90,91]. However, these polymers have shortcomings, including limited mechanical attributes, concerns regarding immunogenicity, variations in quality between batches, and the possibility of contamination with pathogenic impurities (Table 1) [88].

In contrast to natural polymers, synthetic polymers (e.g., polypropylene fumarate, polyanhydrides, poly (orthoesters), poly (phosphazene), and saturated aliphatic polyesters like poly (glycolic acid), poly (lactic acid) (PLA), PCL, along with their copolymers, and poly (glycerol sebacate) (PGS), poly ethylene glycol (PEG)-modified PGS) are intentionally designed and synthesised with precise compositions and structures. This enables consistent production on a large scale and the ability to customise properties such as mechanical strength, porosity, and degradation rate to suit specific requirements. These biodegradable synthetic polymers have gained widespread use as scaffold materials in tissue engineering [88,93,94,97]. However, reports suggest that using chemical cross-linkers and polymerizers in their production could raise concerns about immunogenicity and toxicity [95,96]. Moreover, many of these synthetic polymers exhibit hydrophobic properties and lack cell-binding domains, making it difficult to promote cell adhesion [88]. Yu et al. [98] synthesized a mechanically robust and flexible membrane consisting of PEGylated PGS (PEGS) coordinated by β-TCP nanoparticles (PEGS/β-TCP) and evaluated its efficacy as a prospective biomaterial for GBR therapy. According to the results, the resultant PEGS/β-TCP composite membranes exhibited a controllable degradation rate and reinforced mechanical properties. A maximum tensile strength of 9.58 ± 0.02 megapascals (MPa) was presented in the P20T50 group (weight ratio of β-TCP to PEGS20 prepolymer is 50%), about 1.5-fold higher than that of P20T0 without β-TCP. For cellular responses, the PEGS/β-TCP membranes showed desirable cell attachment and viability upon rat bone mesenchymal stem cells (rBMSCs). The incorporation of β-TCP definitely enhanced alkaline phosphatase activity and promoted the mineralization, thus facilitating the osteogenic differentiation. The in vivo result in a rat calvarial defect model reaffirmed the favorable bone regenerative ability of the fabricated membranes, especially for P20T50 with the highest bone volume/tissue volume ratio (BV/TV) at both 4 weeks (17.26 ± 1.49%) and 8 weeks (23.24 ± 2.85%) after the surgery. Therefore, the PEGS/β-TCP composite membranes prepared by this prepolymer mixing–in situ crosslinking process will be a prospective biomaterial for GBR therapy [98].

### 4.2. Additive Manufacturing Techniques

Additive manufacturing (AM) encompasses various fabrication methods whereby 3D items are built by adding and processing materials layer by layer, often utilising commercial computer-aided design tools [99,100]. This approach allows for creating bone scaffolds featuring accurately specified inner and outer configurations. The American Society for Testing and Materials and the International Standards Organization have systematically divided the AM process into seven different categories, each of which includes a wide range of suppliers, solutions, and material choices [101]. These categories are: Sheet Lamination (SL), Directed Energy Deposition (DED), Material Extrusion (ME), VAT Photopolymerization (VP), Material Jetting (MJ), Binder Jetting (BJ), and Powder Bed Fusion (PBF). It is crucial to remember that terminology in the AM industry is flexible; for instance, the PBF process may also be referred to as direct metal laser sintering, selective laser sintering (SLS), or selective laser melting. In a similar vein, ME is frequently referred to as fused filament fabrication or fused deposition modeling (FDM). Although digital light projection (DLP) is also used in the VP class with a DLP projector as the light source, stereolithography, the pioneer in AM, is frequently used in this class. The MJ category is linked to brand names like PolyJet and NanoParticle Jetting. BJ is also referred to as ColorJet printing or 3D printing. Laminated object manufacturing and ultrasonic AM are both SL processes. DED procedures include electron beam AM and laser-engineered net shaping. There are also many additional methods and technologies in the dynamic AM environment [101]. Some AM techniques, their principles, basic features, and examples of these technologies used in GBR application are shown in Table 2. Commonly employed AM methodologies encompass 3D printing, FDM, and SLS.

#### 4.2.1. 3D Printing

Three-dimensional printing is a construction technique that utilises various materials, such as ceramics, powders, plastics, metals, liquids, or even living cells as bio-inks. This method builds a 3D structure by adding these materials layer by layer in a sequential manner. Bio-ink characteristics, such as viscosity, gelation, and cross-linking, hold significant importance in shaping the quality and morphology of the printed items. These characteristics also protect the objects during printing, influencing cell adhesion, viability, and growth [119]. Ultimately, a 3D model is created as the bio-ink solidifies under the guidance of a 3D modelling program linked to a computer [120].

#### 4.2.2. Fused Deposition Modelling

**Figure 4:**
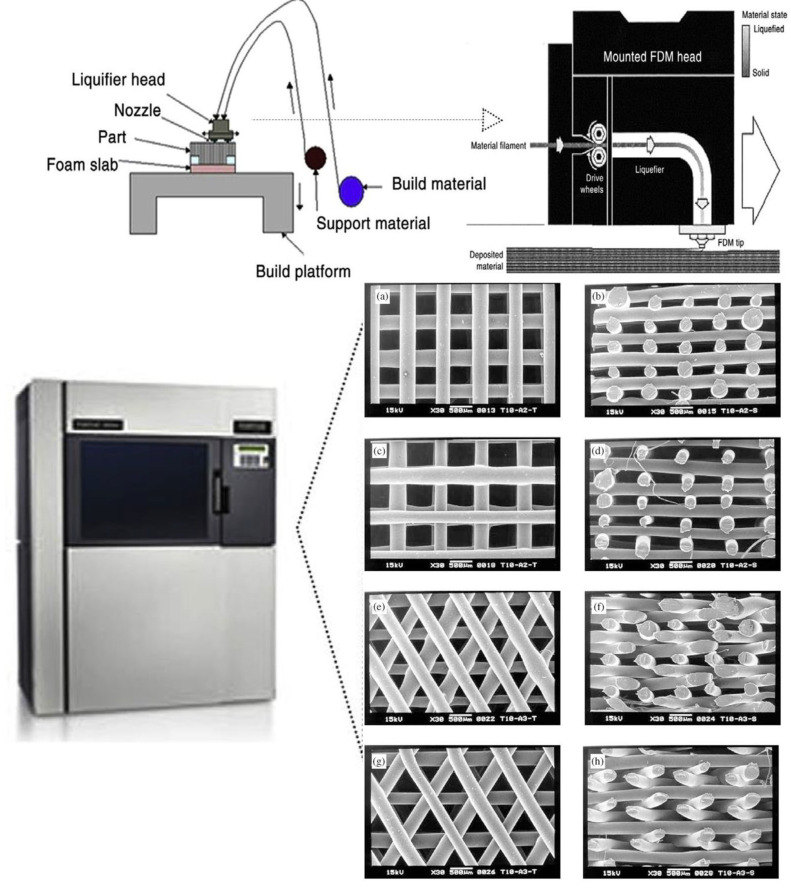
(**a**–**h**) A schematic representation illustrating the printing procedures involved in fused deposition modelling is depicted. Reproduced with permission from Ref. [122]. Copyright 2002 Elsevier, Amsterdam, The Netherlands and [123]. Copyright 2014 Elsevier, Amsterdam, The Netherlands.

FDM represents a solvent-free manufacturing method that employs a 3D AM process centred on extrusion (Figure 4). This technique produces scaffolds characterised by enhanced dimensional precision and product excellence within a reduced time frame.

Within this approach, a thermoplastic substance is placed in a slender layer using a temperature-regulated extruder, assembling the scaffold through a stepwise layering process [119,121]. The precision of the FDM structure is impacted by variables like nozzle diameter and the category of polymer material used. FDM generates scaffold structures that are significantly porous, possessing regulated porosity. It is frequently utilised for fabricating surgical guides, implants, and prostheses. Nevertheless, the direct printing of cells through the FDM process is unattainable due to the degradation of cells prompted by elevated temperatures and unfavourable pH conditions [119].

#### 4.2.3. Selective Laser Sintering

**Figure 5:**
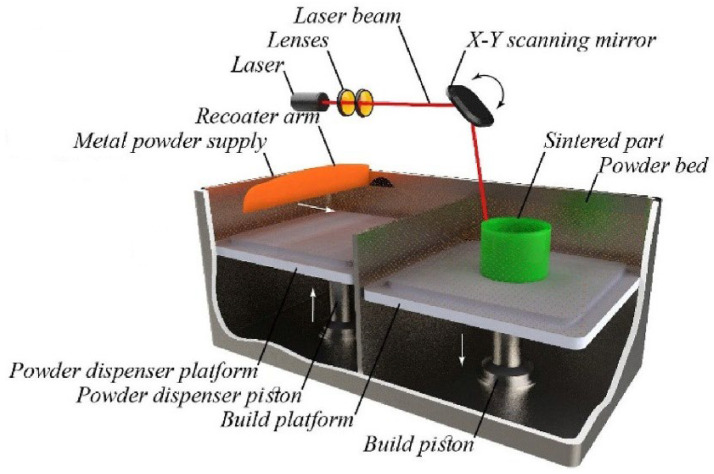
Illustrations commonly depicting the process of Selective Laser Sintering are provided. Reproduced with permission from Reference [125], published by Elsevier in 2023, Amsterdam, The Netherlands.

SLS is a technique that utilizes a high-powered laser beam to elevate the temperature of a material, which can be plastic, metal, ceramic, or glass powder. This operation fuses the powder layer by layer, without melting, resulting in the formation of a 3D structure (Figure 5) [124]. This technique was initially devised by the University of Texas in 1986. Multiple polymers are manufactured utilizing this method, encompassing poly (l-lactic acid) (PLLA), polyvinyl alcohol, polyamide (PA), polyether ether ketone, and PCL. Nevertheless, owing to the elevated temperatures associated with the process, a constraint of this approach is the difficulty in directly integrating viable cells and biomaterials into the scaffold [119].

### 4.3. Other Fabrication Methods for Resorbable Guided Bone Regeneration Scaffolds

#### 4.3.1. Electrospinning

Electrospinning is a technique employed to create nanofibrous scaffolds from a solution by applying high voltage. The application of high voltage induces a charge on the liquid and counteracts the liquid’s surface tension, causing liquid droplets to elongate into nanofibers (Table 3). A standard electrospinning arrangement comprises a spinner equipped with a syringe pump, a high-voltage power source, a metallic needle, and a stationary or rotating grounded collector. Throughout the process, the solvent evaporates and the jet solidifies, resulting in the formation of a nonwoven fibrous membrane [126].

#### 4.3.2. Phase Separation

The phase separation technique is employed to fabricate porous polymeric scaffolds by utilizing variations in thermal energy to trigger the demixing of a specific polymer within two incompatible solvents (Table 3). Solutions containing polymers such as PLLA experience thermodynamic instability at lower temperatures. Upon exposure to higher temperatures, the solution becomes saturated, resulting in the separation of the polymer-rich phase from the solvent-rich phase. Subsequent to this, the specimen is subjected to high-temperature treatment and subsequently rapidly cooled, inducing liquid–liquid phase separation. The polymer-rich phase solidifies or precipitates, giving rise to a significantly porous configuration within the polymer matrix, while the solvent-rich phase is eliminated using methods such as extraction, sublimation, or evaporation [119].

#### 4.3.3. Salt/Porogen Templating

Salt templating is a commonly employed technique for producing porous hydrogel scaffolds, valued for its simplicity and cost-effectiveness. This method involves blending polymer precursors dissolved in aqueous or organic solvents with salt crystals (Table 3). The mixture is subsequently polymerized and/or crosslinked to shape monolithic scaffolds around the salt template. Eventually, the salt is removed from the matrix by washing it with water or weak bases, resulting in the creation of micro/macropores within the hydrogel that mirror the size of the salt crystal template [128].

Sodium chloride (NaCl) salt crystals find extensive use in producing porous hydrogel scaffolds due to their easy accessibility and inertness within biological systems. For example, NaCl templates have been employed to create porous photocrosslinked oligo(polyethylene glycol) fumarate hydrogels, offering the ability to tune pore sizes from 100 to 500 µm [128]. Alternative salts can also function as templates; calcium carbonate (CaCO_3_), for instance, is noteworthy for its minimal solubility under high/neutral pH conditions and increased solubility in acidic surroundings [129]. Sergeeva et al. [129] utilized a CaCO_3_-based templating approach to create stable alginate gels characterized by controlled pore dimensions spanning from 5 to 50 µm. The study investigated the mechanism behind pore formation, considering two influencing factors for pore size: (i) the osmotic pressure generated during the dissolution of sacrificial CaCO_3_ templates, and (ii) the density of the alginate gel network. The findings revealed that osmotic pressure could reach an upper limit of 100 MPa but had no impact on the gel’s porosity. Furthermore, additional osmotic pressure, within the range of kilopascals, induced by dextrans pre-encapsulated within CaCO_3_ vaterite, proved insufficient for enlarging the pores. The stability of pores relied solely on the density of the gel network and the availability of crosslinking calcium ions within a given time. Pores would collapse if the template dissolution occurred too slowly or if there was an insufficient alginate concentration (below 2%). The hydrogels prepared exhibited a relatively soft nature, characterized by a Young’s modulus in the tens of kilopascals range, making them suitable for use as soft porous scaffolds with precisely tuned internal structures [129]. The minimal solubility of CaCO_3_ in high/neutral pH environments and increased solubility in acidic conditions is a crucial property. This property enables the creation of hydrogels under aqueous conditions, preserving the size and structure of the salt template without the need for organic solvents [128].

## 5. Strategies to Enhance Bioactivity and Cellular Response

### 5.1. Materials to Assist Adhesion, Migration, and Proliferation of Osteoblasts

Numerous approaches aim to enhance the effectiveness of GBR substances in facilitating osteoblasts or their precursor cells to adhere, migrate, and proliferate. These substances are classified into four groups: (i) naturally occurring macromolecular materials promoting osteoblast adhesion; (ii) calcium nanoparticle-infused phosphate compounds; (iii) materials containing drugs or GFs; and (iv) materials guided by mechanical conditioning for osteoblasts (Table 4) [2].

#### 5.1.1. Osteoblast-Adhesive Natural Macromolecular Materials

**Figure 6:**
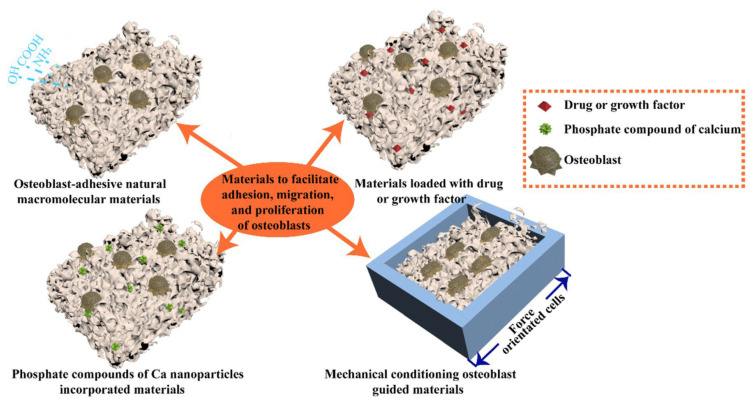
Various adhesive mechanisms used for binding natural macromolecular materials on osteoblast. Reproduced with permission from Ref. [2], Copyright 2022, Elsevier Ltd., Amsterdam, The Netherlands.

GBR substances commonly employ natural macromolecules or their derivatives due to their pronounced attraction for cells. These macromolecules encompass hydroxyl, carboxyl, amino, aldehyde groups, and functional peptides that can engage with membrane proteins or adhere to calcium ions, enhancing the interaction between osteoblasts and the materials. Moreover, specific natural materials resemble bone components, aiding in osteoblast adhesion, migration, and proliferation (Figure 6) [2].

Zhou et al. [131] established a fundamental PLGA/PCL electrospun membrane, which they later coated with collagen I and Ca-chelated polydopamine. The incorporation of collagen into the PLGA/PCL membrane enhanced hydrophilicity, promoted cell adhesion, and facilitated cell penetration. Ca-chelated polydopamine amplified interactions between cells and the material, leading to elevated integrin expression, increased proliferation, and the promotion of osteogenic differentiation in osteoblasts.

#### 5.1.2. Phosphate Compounds with Incorporated Ca Nanoparticles Materials

Bioactive ceramics, such as hydroxyapatite granules and β-TCP, are frequently incorporated into GBR materials. This integration aims to improve the materials’ hydrophilicity, mechanical characteristics, and surface features to promote biocompatibility, osteoblast affinity, proliferation, and mineralization. Furthermore, these ceramics also amplify the osteoinductive and osteoconductive properties of GBR materials [2]. Researchers have explored the amalgamation of PLGA electrospun membranes with hydroxyapatite and β-TCP in several investigations [132,133]. The incorporation of hydroxyapatite and β-TCP has shown the ability to counteract the acidic by-products of PLGA degradation. As a result, the resultant composite membrane, hydroxyapatite-β-TCP (BIL 60:40), demonstrated enhanced mechanical attributes (by 37.8%) and degradation rate (by 22% after 60 days) in comparison to pure PLGA (BIL D95:05). Osteoblast proliferation (by 26.3% on day 7) and migration on membranes containing BIL 60:40 showed notably greater levels compared to the BIL D95:05 group. Undoubtedly, the incorporation of these bioactive ceramics substantially heightened the osteoblastic reaction of the composite membranes, underscoring their promising utility in the realm of bone tissue engineering applications [133].

#### 5.1.3. Materials Loaded with Drugs or Growth Factors

Local drug delivery systems are crucial in enhancing the functionalities of biomaterials used in bone and periodontal tissue engineering. By incorporating drugs and GFs into these delivery systems, researchers can precisely control and modulate the regenerative processes of host bone and periodontal tissues. These systems enable targeted delivery, ensuring that the bioactive substances are released at the intended site and time, promoting specific cellular responses and tissue differentiation. This approach holds significant potential for optimizing the success of bone and periodontal tissue engineering strategies.

The use of BMP-2 in bone regeneration is indeed significant. The injectable BMP-2 formulations based on cross-linked gelatine hydrogel, loaded with magnesium pins, provide controlled and sustained release of BMP-2 over a specific period (for 40 days or more) [134]. This gradual release is synchronized with the hydrogel’s degradation rate, allowing for extended exposure of osteoblasts to the GF. The incorporation of these hydrogels within cannulated screws contributes to delayed biodegradation of the screws, which helps maintain structural integrity while promoting MC3T3-E1 osteoblast cell differentiation (by 27.4%). These enhancements were found to be closely associated with the concentration of BMP-2 present within the hydrogels, in which the hydrogel loaded with 10 µL BMP-2, compared to hydrogel loaded with 5 µL BMP-2, could enhance the cell differentiation by 18.5%. The concentration of BMP-2 within the hydrogel appears to play a critical role in influencing these positive outcomes, highlighting the importance of precise dosage control for optimising bone regeneration [134]. It is without a doubt crucial to incorporate antibacterial efficacy into GBR, especially in the oral and maxillofacial domain where infection risks are high. Different antibacterial agents and tactics have been investigated to improve the antibacterial properties of resorbable GBR scaffolds. These include the incorporation into the scaffold material of antimicrobial substances like antibiotics [135], silver nanoparticles [136], or antimicrobial peptides [137] into the scaffold material. By injecting these substances, scaffolds are given the ability to actively fight infections and promote an environment that is sterile and supportive of the best possible tissue healing. Silver nanoparticles have been successfully incorporated into resorbable scaffolds in several studies [136,138], demonstrating their effectiveness in preventing bacterial colonization on scaffold surfaces. Additionally, studies have explored the controlled release of antibiotics [139] from scaffolds to produce localized antibacterial effects while maintaining a favourable environment for tissue regeneration. These findings highlight the crucial role that antibacterial properties play in GBR scaffolds when it comes to applications in the oral and maxillofacial regions.

#### 5.1.4. Mechanical Conditioning of Osteoblast Growth

Indeed, mechanically conditioning osteoblasts using GBR materials is an intriguing approach. The hierarchical structure of bone, characterised by its specific alignment of mineralised collagen fibrils within osteons, has inspired researchers to leverage surface characteristics of bone scaffolds to influence cell behaviour. By mimicking these structural features in GBR materials, scientists aim to create substrates that guide osteoblast adhesion, proliferation, and differentiation. This concept capitalises on the understanding that mechanical cues play a significant role in cellular responses, and by replicating natural bone’s mechanical environment, it is possible to enhance the performance and effectiveness of bone regeneration materials.

### 5.2. Surface Modifications and Coatings

Alveolar bone’s unique nanostructure, featuring nHA distribution within self-assembled collagen fibrils, is a valuable reference for biomaterial design. By incorporating surface micro or nanostructures and macroporosity into biomaterials, researchers aim to mimic the natural tissue structure of alveolar bone. These biomaterial properties provide a similar topographical environment and influence the local microenvironment at the cellular level. As a result, these designed biomaterials have the potential to modulate the host response and significantly impact tissue healing processes during bone regeneration [140]. Given the potential for optimisation and biomimicry, nanotopography becomes a valuable tool for osteoimmunomodulation aimed at promoting bone healing and regeneration.

The surface properties of biomaterials, including factors like topography and stiffness, can be tailored through various techniques. These techniques encompass mechanical methodologies like micro/nanopatterning, grinding, and blasting, chemical procedures, such as acidic or alkaline treatment, sol–gel processes, anodic oxidation, poly electrolyte multilayer coating, electrochemical anodisation, and spin coating, as well as biological modifications that involve the integration of bioactive molecules. These adjustments aim to augment the interactions between biomaterials and cells, influence host inflammatory reactions, and ultimately enhance the integration and regenerative potential of the biomaterial [141,142].

### 5.3. Mechanisms of Bone Regeneration with Resorbable Guided Bone Regeneration Scaffolds

Creating biomaterials that can replace autologous and allogeneic grafting techniques has made bone repair successful in medical scenarios. Using a biocompatible scaffold during surgery is a prevalent approach to stimulate the development of fresh bone by supporting the migration, proliferation, and differentiation of cells [143]. Craniofacial bone augmentation frequently employs a range of biomaterials, often classified into inorganic and organic types. Inorganic scaffolds often include CaP bioceramics, while organic scaffolds are typically composed of natural or synthetic polymers [144]. Biomaterials aim to mimic living systems or offer similar functions. Critical characteristics for biomaterials intended for tissue replacement include optimal mechanical strength, osteoconductivity, and support for cell attachment and growth [143]. Tissue engineering contributes to bone regeneration by merging the supportive attributes of 3D materials with the combined impacts of osteoinductive agents and recruited stem cells, culminating in advantages for patients [145]. The direct application of bioactive polypeptide GFs onto the root surface can proficiently activate periodontal regeneration by fostering wound healing, subsequently prompting the development of new cementum and connective tissues.

Research on beagle dogs and monkeys has shown successful periodontal tissue regeneration using platelet-derived growth factor (PDGF) and IGF-I [146]. Li et al. [147] conducted an investigation to evaluate the recovery of mandibular defects through the utilization of nHA/PA composite scaffolds incorporating BMP-7 expressing MSCs. Results showed that at 4 and 8 weeks after implantation, animals in Group A (scaffold/MSCs-BMP-7 constructed receivers) exhibited more advanced bone development and mineralization compared to Group B (scaffold/MSCs constructed receivers). Animals in Group B demonstrated greater improvements than those in Group C (control, pure scaffold receivers). However, no significant differences were observed at the 16-week mark. The study concluded that MSCs-nHA/PA composites transduced with BMP-7 significantly accelerated the bone formation process [147].

### Cellular Events during Bone Regeneration with Scaffolds

Bone healing is an intricate process involving a series of molecular and cellular events that are evolutionarily conserved [148]. In the realm of critical size bone injuries, two strategies are currently gaining prominence in clinical applications. One approach focuses on utilizing scaffolds with bioresponsive elements, including osteoconductive and osteoinductive components, to stimulate the inherent cellular environment consisting of immune cells and stem/stromal progenitor cells. This stimulation aids in enhancing the healing and regeneration of bone [148]. The other approach revolves around in situ ectopic cellular reprogramming, achieved through the delivery of transcription and trophic factors, RNA-based therapies, manipulation of epigenetic factors using suitable biomaterials, and even in vivo gene editing [149].

The success of these innovative therapeutic approaches relies on the development of effective scaffolds that possess both strong osteogenic properties and high osteoinductive potential, with the ultimate goal of achieving clinical success. This approach offers an advantage over traditional autografts and allografts by eliminating the complications associated with donor sites [148]. Moreover, through a comprehensive comprehension and assessment of the factors implicated in the bone regeneration process, it becomes feasible to select suitable bioresponsive materials, whether derived from nature or synthesized, that closely emulate the bone microenvironment at the fracture site. This strategic choice, coupled with the prospect of subsequent refinement during scaffold production, will ultimately aid in promoting the process of healing, integration, and remodelling of the newly generated bone. These steps are crucial in bypassing lower therapeutic effectiveness and minimizing potential side effects [150].

## 6. Applications of Metamaterials in Tissue Engineering and Regenerative Medicine

Metamaterials refer to artificially engineered substances designed to possess properties and functionalities not commonly found in natural or conventionally manufactured materials [151]. Among these, triply periodic minimal surfaces (TPMSs) have captured the attention of scientists due to their mathematically controlled, captivating geometries, intricate porous structures, and customizable mechanical characteristics, making them ideal for biomimetic porous scaffold fabrication [152]. TPMSs and their variations have been extensively explored in the scientific literature, primarily because of their prevalence in biological systems [153]. These materials offer remarkable attributes, such as mathematically controllable geometries, highly interconnected porous structures, adjustable mechanical properties, and permeability, which hold significant promise for applications in tissue engineering and regenerative medicine. These advantages facilitate enhanced cell adhesion and growth, seamless tissue integration, efficient fluid and oxygen permeation, and the potential for vascularization [154].

**Figure 7:**
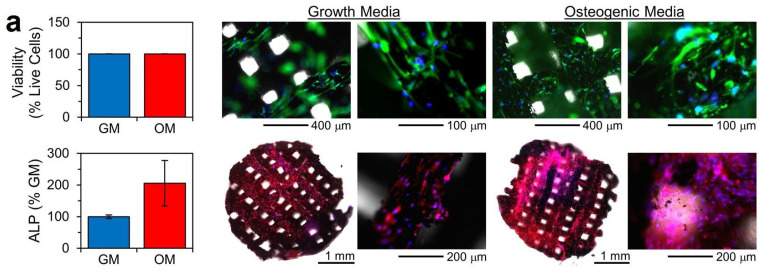

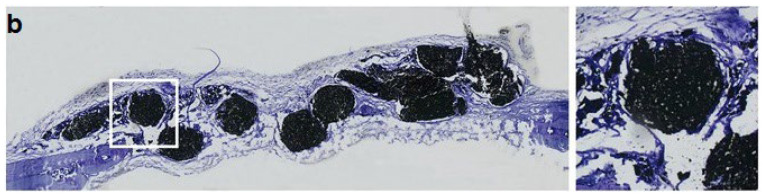
(**a**) Assessment of the biocompatibility and osteogenic differentiation of human mesenchymal stem cells (hMSCs) on structures based on triply periodic minimal surfaces (TPMS). (**b**) Histological images depicting calvarial defects implanted with a bone graft substitute based on TPMS structures. Reproduced with permission from Reference [156], published in Nature in 2022.

In recent years, TPMSs have emerged as a promising option for addressing bone defects, thanks to their resemblance to trabecular bone’s hyperboloidal topography [155]. For example, Daneshmandi et al. [156] conducted a study involving the design, fabrication, and examination, both in vitro and in vivo, of a TPMS-based bone graft substitute. In their in vitro experiments, the scaffolds exhibited cytocompatibility and stimulated the osteogenic differentiation of human MSCs (hMSCs), as indicated by the presence of alkaline phosphatase (Figure 7a). Notably, there were no significant differences in cell viability between growth media and osteogenic media, and no cell death was observed. However, while in vitro data are valuable, they may not always accurately predict the biological response in vivo. To address this concern, the researchers utilized a mouse model featuring a critical-sized calvarial defect to assess the osteogenic effectiveness of the TPMS-based bone graft substitute at an actual bone site. After an 8-week period, the study evaluated the substitute’s capacity, created through 3D printing, to regenerate cranial tissue within this critical-sized calvarial defect (Figure 7b). The histological findings demonstrated that the TPMS scaffold not only promotes cellular ingrowth but also preserves donor cells while triggering osteogenic differentiation. Remarkably, the researchers noted that the TPMS bone graft substitute exhibited in vivo reabsorption and biodegradation, as evidenced by a reduction in matrix mass over time. This natural bio-resorption is an unusual occurrence for synthetic materials and holds significant potential as a resorbable osteoinductive matrix. Importantly, no adverse effects or build-up were observed in vital organs [156].

## 7. Additional Modern Scaffold Design Techniques

In addition to TPMS, a number of additional cutting-edge design methods for scaffolds have surfaced. The solid isotropic material with penalization (SIMP) approach, the Voronoi method, machine learning (ML), genetic algorithm (GA), and AM method (e.g., direct metal laser sintering) are a few of these techniques [157,158,159].

### 7.1. Solid Isotropic Material with Penalization (SIMP) Method

**Figure 8:**
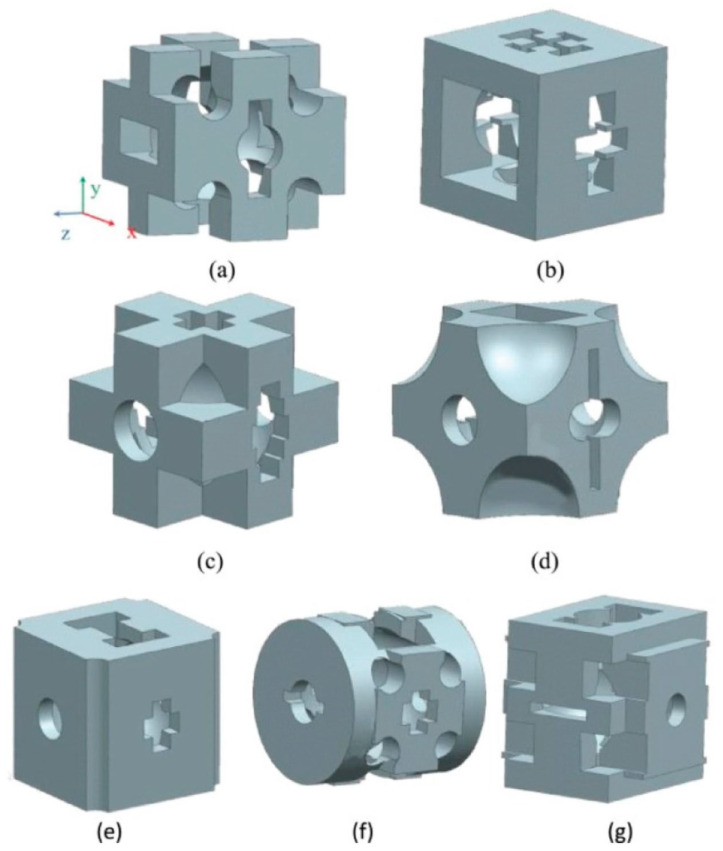
Representations of the optimized cell model at different locations: (**a**) M15; (**b**) M16; (**c**) M17; (**d**) M18; (**e**) M19; (**f**) M20; and (**g**) M21 (numbers designating different locations). Reproduced with permission from Reference [161], Copyright 2021, IEEE Xplore.

The SIMP method is a topology optimization technique. A material interpolation model called SIMP allows for the existence of intermediate relative densities in the 0 to 1 range. To produce precise topological results, material density is penalized and low-density cells are filtered out. This effective technique can be used to create complex structures with multiscale features [160]. Figure 8 shows an illustration of a cell that has been optimized using the SIMP technique.

In order to engineer a 3D structure with gradient porosity similar to the natural pore structure, the SIMP method was used to create a porous structure that took advantage of the unique morphology and mechanical properties of trabecular bone [161]. A homogenization-based algorithm was used in the design of a 3D bone scaffold to achieve the desired porosity and elastic properties [162]. Using well-known biomaterials, the authors also showed that the technique can produce a porous structure that mimics the anisotropic stiffness present in human trabecular bone. This method made it possible to design porous structures with the best permeability properties [163]. Researchers also used this approach to balance the competing properties of stiffness and fluid permeability when designing multifunctional porous material microstructures [164]. To reduce the difference between the optimized scaffold’s effective elastic tensor and that of natural bone, they used topological optimization techniques. Comparative analysis showed that the elastic tensor of the bone scaffold that slightly outperformed that of natural bone was the optimum level for bone remodelling [165]. Although the SIMP method has many benefits, the optimized structures it creates frequently experience numerical instabilities, such as tessellation, grid dependence, and grayscale cells. Furthermore, additional non-physical constraints are needed during the optimization process to ensure pore connectivity in microstructure design (Table 5) [166].

### 7.2. Voronoi Method

A method for modelling erratic open-hole structures used to define spatial regions is the Voronoi tessellation method (VTM). For the purpose of building scaffolds for bone regeneration, previous studies have developed a method that enables the design of interconnected porous lattices that mimic particular tissue characteristics (Table 5) [157]. With this technique, the porosity and pore size of the scaffold are adjusted to match the anatomical shape of the defect. Its main benefit is the introduction of geometrical heterogeneity, which produces highly bioinspired shapes.

Based on the Voronoi structure design principles, a parametric design approach for lattice porous structures has been created [167]. Due to the stable distribution of seed points within the lattice cells, the method ensures variations in model porosity and surface area. Since each cell’s porosity can be customized using this method, lattice structures with uniformly fractionated or graded porosity distribution can be created. To manage the scaffold’s dominant elastic modulus and lessen stress shielding between the scaffold and bone, a structural design method based on VTM has been suggested [168]. This technique can enhance stress shielding while producing a gradient scaffold that matches the natural bone modulus. The nodal connectivity Z, strut density d, and strut thickness t parameters can each independently define the stochastic structure during the design phase. These parameters can also be used to predict relative density, stiffness, and ultimate strength. The benefit of the stochastic structure is that it allows for the incorporation of property gradients within the same component, as well as accommodating a variety of design requirements in a single integrated model [157].

In irregular porous structures, the relationship between porosity or apparent elastic modulus and compressive strength is not simple, because changing one parameter may cause the other to change as well. Further research is necessary to fully understand this intricate interaction, which results from the intricate irregular porous structure of VTM [169].

### 7.3. The Machine Learning Approach

ML has become a useful tool in a variety of research fields. This area of artificial intelligence (AI) is excellent at finding patterns in large datasets and is crucial for a variety of tasks, such as spam detection, drug discovery, and speech and image recognition [157]. The design of new materials with desired properties is made possible by ML algorithms, which also allow for the prediction of material and structural properties.

In order to lessen proximal femoral stress shielding, ML techniques (MLTs) have been combined with parametric finite element analysis (FEA) to improve the optimization of the geometry of short-stemmed hip scaffolds [170]. In order to achieve the best geometry, a minimization algorithm has been used, allowing for the exploration of hip brace geometry parameter values that had not previously been considered. Costs associated with computation have been significantly reduced by combining FEA, MLT, and search pattern optimization algorithms (Table 5) [170]. To cut down on computation time, an effective method for designing scaffolds in biological tissues has been suggested [171]. This approach uses a probabilistic model of mesoscale cortical bone to solve the optimization problem of titanium scaffold geometry. A difficult constrained nonconvex optimization problem in biomechanics can be handled by this cutting-edge algorithm under uncertain circumstances. ML has been used in a novel way to design layered materials [172]. It uses a database of FEA structures totalling hundreds of thousands of structures for training and incorporates a self-learning algorithm that filters out inferior designs to find the best candidates. This method demonstrates the potential for replacing detailed microstructural data with ML, enabling material analysis and design. In comparison to traditional approaches, this paradigm can result in the discovery and creation of new materials with significantly higher computational efficiency. The Inverse Homogenization (IH) mapping from attributes to cell shapes can also be learned using a Generative Adversarial Network (GAN) model, which can then be used to optimize functionally graded cell structures [173]. Using input parameters like tensile modulus, elongation at break, and tensile strength of natural cartilage, ML algorithms have also been used to predict the most suitable polymer/blends for replacing cartilage [174].

It is important to keep in mind, though, that the time needed for MLT can increase exponentially as the number of parameters rises. Data-driven models, such as IH-GAN, have limitations because they are only able to generate cell shapes and properties within a given training data distribution, which may limit the effectiveness of the optimized cell structure [173].

### 7.4. The Genetic Algorithm (GA)

Due to their high efficiency (Table 5), genetic algorithms (GAs) are frequently used in structural optimization designs [175]. As a result, GAs are frequently used in current research projects to create the best scaffold structures. To design scaffolds, for instance, a novel computer-based method that combines FEA and generalized additive modelling has been developed. This method chooses the scaffold fibre diameters and inter-fibre spacing to achieve the necessary stiffness for each degradation stage [176]. With the help of the Non-Dominated Sorting Genetic Algorithm II and the Kriging method, hierarchical 3D porous structures with the best crush resistance have been designed to perfection [177]. An inverse model based on the multi-constrained knapsack problem was solved using a hybrid GA to address the porous scaffold’s complex structure. The resulting biomimetic bone scaffold showed superb bioactivity, enhanced mechanical attributes, and a predictable rate of degradation [178]. An asymptotic homogenization scheme and a GA are combined in a numerical method for metamaterial reverse engineering to find the ideal internal material pattern using the entire range of parameters found in the target compliance tensor [179]. For cementless femoral scaffolds, a novel custom shape optimization scheme was created by combining FEA with multi-objective GAs [180]. This optimization framework, which was based on a GA capable of handling multi-objective optimization problems with nonlinear constraints, was created to automatically generate preoperative planning solutions. Primary stability was improved over the original design thanks to the GA-driven optimization of the scaffolds, which was carried out to minimize predicted micromotion according to the back-propagation neural network [157].

Although effective, the GA has computational time limitations, especially for scaffolds with complex structures [157]. In contrast to current mono- and multi-scale optimization techniques used in orthopaedic applications, Smit et al. [159] introduced a full-scale topology optimization approach for optimizing synthetic bone scaffolds over multiple length scales. The findings showed that, with an 81% improvement over the multi-scale approach, the porous scaffold structure could be fine-tuned to achieve desired morphological properties for enhanced bone in-growth. However, more study is required to determine its effect on clinical applications [159]. A method for designing graded porous structured acetabular implants was presented by Mukherjee et al. [158] along with parameters that could be used to create models using AM (direct metal laser sintering). In order to maintain gradation continuity, this design method relied on slice-wise modifications, and it used a geodesic dome-type design to create the acetabular cup model. In terms of porosity and pore size, the results showed that they were nearly in line with the intended values. Additionally, the stiffness, compression testing, and compliant bending-dominated behaviour of the structures closely matched the characteristics of human trabecular bone. Finally, the authors suggested that the best implant design may require site-specific bone in-growth studies [158].

## 8. Applications of Artificial Intelligence in Tissue Engineering and Regenerative Medicine

Despite the long history of AI, recent advances in ML, deep learning, and natural language processing have ushered in a new era of more advanced AI systems. Researchers now have the ability to sift through enormous datasets, spot intricate patterns, make data-driven predictions [181], and even learn from their mistakes by changing their behaviour without explicit programming thanks to ML. ML is used in a variety of fields, such as biomedical engineering [182], autonomous vehicles [183], and image recognition [184].

Artificial neural networks are used in deep learning, a branch of ML, to learn from data. These neural networks can recognize complex patterns and make decisions based on their training data because they are created to mimic the structure and operation of the human brain [185,186]. In the field of AI, deep learning has changed the game by allowing machines to complete tasks previously thought impossible. Its ability to manage complicated and large datasets is one of its key strengths [187]. With data that are too numerous or complex for human processing, traditional ML algorithms frequently struggle. Deep learning algorithms, on the other hand, can analyse millions of data points and find patterns that a human might miss [181]. The ability of deep learning to learn and advance over time is another remarkable quality. Traditional ML algorithms frequently have memory issues, necessitating manual parameter adjustments in order to improve performance. Long Short-Term Memory and recurrent neural networks are two examples of deep learning algorithms that can autonomously adapt in response to the data they process. As they encounter more data, deep learning algorithms can thus continuously improve and develop [181,188].

Regenerative medicine is a rapidly advancing field focused on restoring or replacing damaged or diseased tissues and organs, employing cutting-edge technologies like stem cell-based therapies, gene therapy, and tissue engineering. Regenerative medicine offers hope for patients coping with a range of conditions, from heart disease to diabetes and neurological disorders, and has the potential to revolutionize medical treatment. However, efficient regenerative therapies demand the analysis of large and complex data, which is where AI is crucial [181].

AI has emerged as a crucial component for conducting computational simulations and in silico studies within the realm of medical applications. It offers several advantages, including cost-effectiveness and quicker results when compared to conventional medical investigation methods, such as clinical trials and laboratory experiments [181]. At present, numerous ongoing initiatives seek to integrate AI into a broad spectrum of fields, encompassing but not limited to medicine, pharmaceuticals, and healthcare [181,189]. These endeavours are geared towards harnessing the capabilities of AI to optimize and streamline various processes, such as drug development, disease diagnosis, and medical treatment. Through the integration of AI, researchers and healthcare practitioners aspire to achieve heightened accuracy and efficiency, ultimately enhancing the quality of life for both individuals and communities [181,189]. In particular, deep learning plays a vital role in expediting the advancement of regenerative therapies by streamlining tasks, such as the analysis of extensive datasets containing molecular and genetic information. It excels at recognizing patterns and correlations that might elude human researchers, contributing to a deeper comprehension of the fundamental disease mechanisms. Ultimately, this aids in the development of more potent and targeted therapies to combat these mechanisms effectively [181].

The success of tissue engineering methods relies significantly on their capacity to create effective scaffolds capable of nurturing cell growth and differentiation into functional tissue [190]. Scaffolds can be crafted from a diverse range of materials, including ceramics, synthetic polymers, and natural biopolymers, and they can be tailored to emulate the characteristics of native tissue [65]. AI comes into play by optimizing material properties for particular applications, thoroughly analysing how these materials interact with biological systems. This knowledge serves as a foundation for designing and producing scaffolds tailored to specific tissue engineering objectives. The fabrication of scaffolds employs various techniques, which depend on the material type and the desired scaffold properties [191]. AI can indeed play a pivotal role in selecting the most efficient and effective method for fabricating scaffolds tailored to a particular application in tissue engineering. AI algorithms excel in sifting through extensive datasets encompassing various materials and fabrication techniques, pinpointing suitable combinations for a specific purpose. Furthermore, these algorithms can simulate the fabrication process and predict the resulting scaffold’s properties, aiding researchers in optimizing the design while reducing fabrication time and costs. Moreover, AI contributes to quality control by real-time monitoring of the fabrication process, swiftly detecting any deviations from the intended parameters. This ensures that the scaffold aligns with the desired specifications and maintains high quality standards [181].

**Figure 9:**
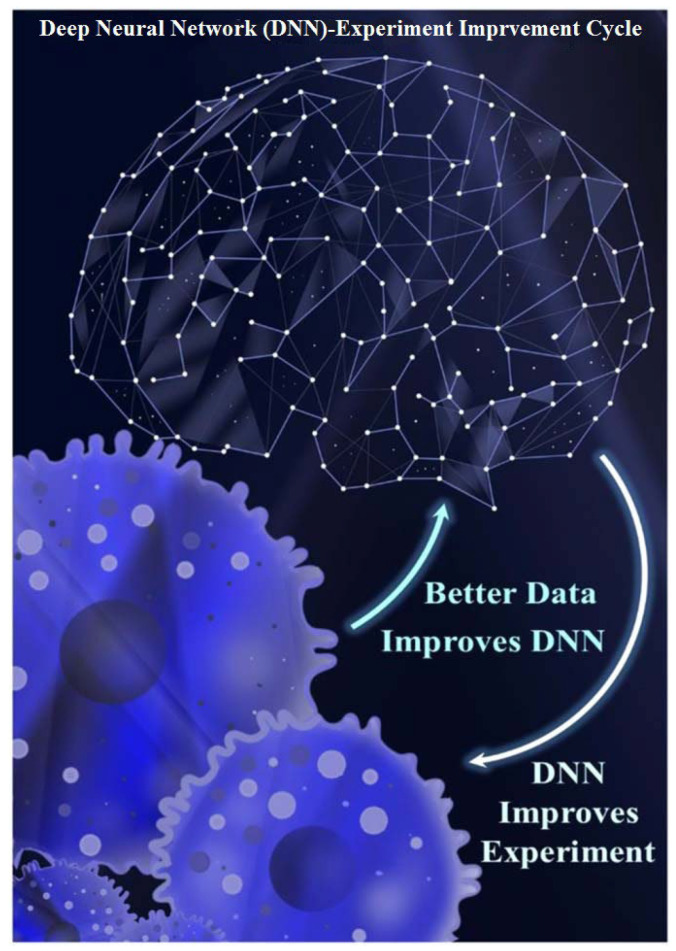
Potential for ground-breaking medical advancements through a collaboration between medical professionals, researchers, and deep learning technology. This potential extends to fields, such as tissue engineering, where numerous uncertainties and challenges, such as parameter issues, currently impede traditional manual experimentation. Reproduced with permission from Reference [192], Copyright 2021, IOPScience, Bristol, UK.

In general, tissue engineering is a key component of regenerative medicine, and its significance continues to grow each year due to aging populations worldwide. While there have been notable advancements in recent decades, several hurdles remain, particularly in the areas of biomaterial design and comprehending the behaviours of stem cells. Nevertheless, by incorporating deep neural networks into both experimental research and clinical applications, it is possible to address many of the current medical challenges. This could pave the way for tailored solutions specific to individual patients and their unique medical conditions (Figure 9). It is plausible that AI may shape the future of bone regeneration [192].

## 9. Conclusions

This literature review provides a comprehensive overview of resorbable scaffolds in bone tissue engineering. It discusses scaffold design, fabrication techniques, materials (including natural and synthetic polymers), and advanced manufacturing methods, such as 3D printing. In addition, this literature review highlights the importance of surface modifications to mimic native bone structures and their impact on cellular responses. Moreover, it explores the mechanisms underlying bone regeneration, including the interplay between bioresponsive scaffolds, GFs, immune cells, and stromal progenitor cells. This paper highlights how these principles are applied in clinical settings to promote integration, healing, and regeneration. Furthermore, this literature review highlights emerging areas of metamaterials and AI applications in tissue engineering and regenerative medicine. Overall, the potential of combining various aspects of material science, manufacturing, and cellular biology is demonstrated to advance the field of bone tissue engineering for improved therapeutic outcomes and patient well-being.

## Figures and Tables

**Table 1:** The features of biodegradable natural and synthetic polymers used for guided bone regeneration.

Type of Polymer	Advantages	Limitations	Ref.
Natural (e.g., collagen, chitosan, gelatine, silk fibroin, alginate, cellulose, and starch)	Excellent biocompatibility, biodegradability, bioavailability, strong cell adhesion, and favourable conditions for cell growth. Can be used alone or in combination to achieve a scaffold with favourable mechanical properties suitable.	Limited mechanical attributes, immunogenicity, variations in quality between batches, and the possibility of contamination with pathogenic impurities. Challenges for vascularization and their integration into the host tissues.	[51,90]
Synthetic (e.g., polypropylene fumarate, polyanhydrides, poly (orthoesters), poly (phosphazene), and saturated aliphatic polyesters like PGA, PLA, PCL, along with their copolymers, PGS, PEG-modified PGS	Can be synthesized with precise compositions and structures; thus, their production can be scaled up and produced with customised properties, such as mechanical strength, porosity, and degradation rate to suit specific requirements. Infinite variety of forms.	Immunogenicity and toxicity. Hydrophobicity and lack of cell-binding domains that reduce their ability to promote cell adhesion.	[88,92,93,94,95,96,97]

PGA: poly (glycolic acid); PLA: poly (lactic acid); PCL: polycaprolactone; PGS: poly (glycerol sebacate); PEG: poly ethylene glycol.

**Table 2:** Additive manufacturing techniques, their basic principles, features, and examples used in guided bone regeneration application.

Class	M.M	Operation	Pros and Cons	Example
Extrusion	FDM	The material undergoes heating until it reaches a molten state and is then extruded through a printing head, employing pressurized extrusion, screw-based extrusion, or a combination of both methods. Upon contact with the base plate or the preceding layer, the extruded material undergoes solidification, giving rise to a filament often referred to as a “strut” [102].	Pros: low cost, ease of use and products with good thermal and mechanical properties [103]. Cons: generally limited its application to PCL and PLA due to its requirement of a thermoplastic [104].	Fabrication of mPCL-TCP scaffolds combined with a dose of 2 mg rhBMP-7 delivered in PRP [105].
	DIW	To produce high-resolution scaffolds, a polymeric ink or binder is extruded. The resulting objects are initially soft and delicate, necessitating the concurrent printing of support materials. Following printing, a post-processing sequence involving drying, de-binding, and sintering is imperative to achieve the desired mechanical properties and structural integrity [102].	Pros: fast printing speed, easy operation, low cost, good printing accuracy, and can be widely applied to various material systems [106]. Cons: lower printing accuracy compared to that of SLA printing technology [106].	Fabrication of bioactive 6P53B glass scaffolds with superior mechanical strength (compressive strength: 136 ± 22 MPa) [107].
Polymerization by light	CLIP	It consists of a bath housing a photopolymer resin and a transparent windowpane. The resin is cured layer by layer using an ultraviolet light beam, while the object is extruded vertically at a consistently slow pace. A nonpermeable oxygen membrane, positioned between the windowpane and the resin bath, enables a continuous laser process to take place [108].	Pros: no lamination typically seen in standard layer by layer SLA polymerization; formation of microscale features with z-axis print speeds up to 1000 mm/h [109,110]. Cons: limited number of commercially available photopolymerizable resins to produce biocompatible products. Mechanical properties of traditional photopolymerized resins are also known to be generally poor [111].	Fabrication of 3D objects of n-HA filled polymeric biomaterials with a high compression strength of 6.5 ± 1.4 MPa [112].
	SLA	This system consists of a bath with a transparent windowpane containing a photopolymer resin. An ultraviolet light beam cures the resin layer by layer while the object is extruded vertically at a steady, slow speed. After each layer is cured, a blade component, filled with resin, sweeps across the windowpane, supplying the necessary resin to solidify the subsequent printing layer [113].	Pros: versatility and the highest resolutions; 5–300 µm, accuracies and the smoothest surface finish. Cons: limited by their ability to be processed into a photo-crosslinkable hydrogel, modified by the addition of photo-crosslinkable groups along the molecular chain [104].	Fabrication of scaffold containing lentiviral gene constructs—Lv-BMP/GFP with dramatically increased expression of osteogenesis marker genes [114].
Powder bed	3DP	An inkjet head dispenses a liquid fusing substance that binds particles within the powder bed. Once a single layer is finished, a new layer of powder is added atop the completed one, and this process is iteratively repeated, layer by layer, until the component is fully constructed [115].	Pros: high fidelity and finite element analysis along with applicability to various materials [104,116]. Cons: limited only in that the material must be in a powder form [104].	Fabrication of scaffolds containing urethane-based PEGylated PGS in ceramic bio-inks with enhanced me-chanical strength and hyperelasticity, and supporting cell proliferation and osteogenic differentiation [116].
	SLS	In the SLS process, a high-powered pulsating carbon dioxide laser is directed onto a bed of powdered material, which has been preheated to just below its melting point. This laser binds the particles together. Similar to other powder bed technologies, SLS necessitates the successive deposition of a fresh layer of powdered material to cover the previously completed cross-section, and this iterative process continues until the 3D object is fully formed [117].	Pros: its capability of producing highly detailed prints with thin walls. Cons: comparison to the other AM techniques, it has a poor dimensional accuracy of just 150–180 µm. Other issues that are associated with SLS include the inability to incorporate growth factors and cells during printing, as well as shrinking and warping of the scaffold due to thermal distortion. Also, natural polymers cannot be utilized in this technique because of the high temperatures that are generated by the laser [104].	Fabrication of customised bioceramic implants to produce bone replacement components [118].

M.M: manufacturing method; FDM: fused deposition modelling; PCL: polycaprolactone; PLA: poly (lactic acid); mPCL-TCP: polycaprolactone-tricalciumphosphate; rhBMP-7: recombinant human bone morphogenetic protein-2; PRP: platelet-rich-plasma; DIW: direct ink writing/robocasting; SLA: stereolithography; CLIP: continuous liquid interface production; 3D: three dimensional; n-HA: nano-hydroxyapatite; MPa: megapascal; Lv: lenti-human cytomegalovirus (CMV); BMP: bone morphogenetic proteins; GFP: green fluorescent protein; 3DP: powder bed and inkjet head 3D printing; PEG: poly ethylene glycol; PGS: poly (glycerol sebacate); SLS: selective laser sintering; AM: Additive manufacturing.

**Table 3:** Step-by-step process of scaffold fabrication using electrospinning, phase separation, and salt/porogen templating methods.

Fabrication Method	Scaffold Development in a Step-By-Step Manner	Ref.
Electrospinning	Charging a liquid under a condition of sufficiently high voltage, resulting in overcoming the liquid surface tension, and subsequently elongation of liquid droplets to nanofibers.	[126,127]
Phase separation	Polymer solutions become thermodynamically unstable at low temperatures; solutions are saturated by increasing the temperature and, subsequently, separated into polymer- and solvent-rich phases. Subjecting the phases to a high temperature and then quenching to result in a liquid–liquid phase separation. A highly porous structure in the polymer matrix is obtained by solidification or precipitation of the polymer-rich phase.	[119]
Salt/porogen templating	Dissolving the polymer precursors in aqueous media or, less commonly, an organic solvent and then mixing with salt crystals. Subsequently, polymerizing and/or crosslinking the dissolved polymer precursors to form monolithic scaffolds around the salt template. Leaching the salt from the matrix (typically using water or weak bases) and creating micro/macropores within the scaffold, which are matched to the size of the salt crystal template.	[128]

**Table 4:** Aims and examples of materials used in guided bone regeneration substances.

Materials	Aim	Example
Naturally occurring macromolecular materials promoting osteoblast adhesion	Increasing the affinity between osteoblasts and materials	Natural macromolecules containing the hydroxyl, carboxyl, amino, or aldehyde groups and functional peptides [2].
Calcium nanoparticle-infused phosphate compounds	Facilitating the biocompatibility, osteoblast affinity and proliferation, and mineralization of GBR materials through enhancing their hydrophilicity, mechanical properties, and topographies. Also enhancing the osteoinductivity and osteoconductivity of GBR materials	Bioactive ceramics, such as hydroxyapatite granules and β-TCP [2].
Materials containing drugs or GFs	Aiding in controlling the complex and self-regenerative phases of the host bone and periodontal tissue, inducing a specific cellular response or differentiation	BMP-2, PDGF, dexamethasone, and alendronate sodium [2].
Materials guided by mechanical conditioning for osteoblasts	Improving the mechanical conditioning of osteoblasts to improve their adhesion, proliferation, and differentiation	Anisotropic microgrooved collagen membranes [130].

GBR: guided bone regeneration; β-TCP: beta tri-calcium phosphate; GFs: growth factors; BMP-2: bone morphogenic protein 2; PDGF: platelet-derived growth factor.

**Table 5:** Comparison of various fabrication methods for the synthesis of scaffolds.

Fabrication Method	Advantages	Limitations	Ref.
SIMP	Appropriate mechanical characteristics	Expensive calculation, complex programming, low-speed calculation	[157,166]
Voronoi	Excellent structure, appropriate distribution of voids	Complex structure, complex relationship between parameters	[157]
ML	Inexpensive calculation	High demand for training data	[157]
GA	Appropriate scalability, simple process, rapid convergence	Complex programming, high experience requirement for parameter selection, slow speed	[157]

SIMP: solid isotropic material with penalization; ML: machine learning; GA: genetic algorithm.

## Data Availability

Not applicable.
